# The Diagnostic Value of Whole Blood lncRNA NR_120420 for Acute Ischemic Stroke

**DOI:** 10.1155/2022/1167394

**Published:** 2022-02-23

**Authors:** Chunou Tian, Zifu Li, Sisi Li, Lei Zhang, Dongwei Dai, Qinghai Huang, Yu Zhou, Bo Hong

**Affiliations:** ^1^Neurovascular Center, Changhai Hospital, Naval Medical University, 168 Changhai Road, Shanghai 200433, China; ^2^Department of Neurosurgery, The First Naval Hospital of Southern Theater Command of PLA, 40 Third Haibin Road, Zhanjiang, 524005 Guangdong, China

## Abstract

**Objective:**

Current clinical practice based on CT or multimodal images to diagnose ischemic stroke always led to substantial treatment delay. We perform this study to explore possible circulating lncRNA biomarker to help promptly diagnose the disease.

**Methods:**

We used microarray to identify the differentially expressed lncRNAs in the peripheral whole blood between AIS patients and controls and verified the results by quantitative polymerase chain reaction (qPCR). Multivariate logistic regressions were performed to determinate the lncRNAs independently associated with AIS occurrence. The ROC curve was used to detect the diagnostic accuracy of candidate lncRNAs in AIS and AIS subtypes, which was classified according to the Oxford Community Stroke Project (OCSP) criteria.

**Results:**

The microarray analysis screened out 5686 differentially expressed lncRNAs. Among the nine selected lncRNAs verified by qPCR, NR_120420 (OR 1.29, 95% CI 1.02-1.65, *P* = 0.037) was found independently associated with AIS after balancing patient baseline characteristics. The receiver operating characteristic (ROC) analysis concerning NR_120420 in total anterior circulation infarction subgroup showed that the area under the curve was 0.86 (95% CI: 0.73-0.99, *P* = 0.003), and at the optimal cutoff point of 1.93, the sensitivity and specificity reached 85.7% and 84.6%, respectively.

**Conclusion:**

Our study indicated that NR_120420 could predict the total anterior circulation infarction with high sensitivity and specificity and could be potentially used as a biomarker for total anterior circulation infarction in AIS patients.

## 1. Introduction

Nowadays, thrombolysis and thrombectomy become standard treatment of acute ischemic strokes (AIS) [1–5]. However, patients' outcomes are closely related to onset to reperfusion time. Current clinical practice based on CT or multimodal images to diagnose the diseases always led to substantial treatment delay, i.e., patients with suspicious stroke have to be sent to hospital and perform CT before initiating such reperfusion treatment. Attempts for faster and more convenient diagnosis are needed to shorten the treatment delay and improve patient outcome.

It is speculated that, in AIS, many molecules and proteins released from injured or dead brain cells may escape from the brain via the disrupted blood-brain barrier and enter the blood flow, which may provide a new approach for the prompt diagnosis of AIS [6–9]. In recent years, the long noncoding RNAs (lncRNAs) have been used in the diagnosis of many diseases [10–13]. lncRNAs are a subtype of noncoding RNAs (ncRNAs) that make up the largest portion of the transcriptome [14] and are involved in many cellular processes after stroke including hypoxia, excitotoxicity, inflammation, oxidative stress, and apoptosis [15]. In such circumstance, some circulating lncRNAs may play important roles in early diagnosis of the AIS [16]. However, such studies are still limited.

In this study, we identified differentially expressed lncRNAs in the peripheral whole blood from AIS patients using microarray and verified them by quantitative polymerase chain reaction (qPCR); we aimed to explore the relationship between circulating lncRNA expression and the classification of AIS.

## 2. Materials and Methods

### 2.1. Patients

The patients with AIS admitted to the Neurovascular Center of Shanghai Changhai Hospital between January 2016 and November 2016 were enrolled in the study. The written informed consent was obtained from these patients. They were selected following the inclusion and exclusion criteria. Inclusion criteria were as follows: time duration from the onset of stroke to confirmation was less than 6 hours. Exclusion criteria were as follows: immune disease, trauma, organ failure, tumor, and infection. The control (CTRL) population was made up of healthy volunteers. The study was approved by the Medical Ethics Committee of Shanghai Changhai Hospital, and all the experiment procedures were carried out according to the 1964 Helsinki declaration and its later amendments or comparable ethical standards.

### 2.2. Blood Collection and RNA Extraction

The peripheral whole blood of AIS patients and CTRLs was collected and added into PAXgene Blood RNA Tubes (BD, New York, America). Subsequently, the blood collection tube would be rapidly transferred to a refrigerator whose temperature was set at -80°C. Therefore, the total RNA was extracted with a PAXgene Blood miRNA Kit (Qiagen, Nordrhein-Westfalen, Germany) from the peripheral whole blood according to the manufacturer's instructions. The clinical data of AIS patients and CTRLs was obtained through clinical or outpatient follow-up.

### 2.3. lncRNA Microarray Hybridization

The Agilent Human lncRNA microarray (4∗180K V5, Design ID: 076500) was used in this experiment. Total RNA was transcribed into double-strand cDNA and then synthesized into cRNA and labeled with Cyanine-3-CTP. After washing, the arrays were scanned by the Agilent Scanner G2505C (Agilent Technologies, CA, USA). Lastly, Feature Extraction software (version 10.7.1.1, Agilent Technologies, CA, USA) was used to analyze array images to get raw data and GeneSpring (version 13.1, Agilent Technologies, CA, USA) was employed to finish the basic analysis with the raw data. To begin with, the raw data was normalized with the quantile algorithm. Differentially expressed genes or lncRNAs with an arbitrary fold change (FC) (≥2) and a *P* value (<0.05) were selected.

### 2.4. Quantitative PCR

A two-step reaction process was used for qPCR (reverse transcription (RT) PCR and real-time PCR). PrimeScript™ RT reagent Kit with gDNA Eraser (Takara-Bio, Shiga, Japan) was used in the first step because it can eliminate genomic DNA. QuantiNova SYBR Green PCR Kit (Qiagen, Nordrhein-Westfalen, Germany) was used in the second step. At the end of the PCR cycles, the sequencing work was performed by a 3730XL sequencer (Applied Biosystems, California, CA, USA) to validate the specific generation of the expected PCR product. The cycle threshold (Ct) was defined as the cycle number when the fluorescence has just exceeded the given threshold. The operators who detected the lncRNAs in blood were blinded to clinical data of the samples. Relative expression levels of lncRNAs were normalized to *β*-actin and were calculated by the 2^-*ΔΔ*CT^ method in triplicate.

### 2.5. Correlation Analysis between lncRNAs and Stroke

Multivariate logistic regressions were performed to evaluate the relationship among candidate lncRNA expression, clinical features, and AIS status. We used ROC curves to clarify the diagnostic accuracy of candidate lncRNAs for AIS.

### 2.6. Correlation between Specific lncRNAs and Stroke Classification

AIS patients were classified according to the OCSP criteria by two independent examiners, namely, total anterior circulation infarct (TACI), partial anterior circulation infarct (PACI), posterior circulation infarct (POCI), or lacunar infarct (LACI), based on their maximum neurological defects [17]. The expression levels of specific lncRNA between AIS patients in different classifications and CTRLs were analyzed statistically.

### 2.7. Statistical Analysis

A *χ*^2^ test was used to compare the qualitative data. One-way analysis of variance (ANOVA) was applied when the quantitative data could follow the normal distribution (the Shapiro-Wilk test, *α* = 0.1) and the homogeneity of variance (the Levene test). The Mann–Whitney test or the Kruskal-Wallis test was performed when the quantitative data did not fit the normal distribution or the homogeneity of variance. All statistical calculations were performed with the SPSS software 21.0. The final data was expressed as mean ± standard deviation (SD), and a value of *P* < 0.05 was considered as significant.

## 3. Results

### 3.1. Patient Characteristics and AIS Classification

Overall, 45 AIS patients and 42 controls were enrolled in this study.  Table 1 shows the clinical baseline characteristics of enrolled patients in the AIS and CTRL groups, respectively. Baseline characteristics such as age, gender, risk factors of ischemic stroke, vital signs, blood glucose, and lipid are balanced between the two groups.

Six AIS patients and three controls were selected randomly (simple random sampling, random number table) for microarray detection. 39 AIS patients and 39 controls were selected for subsequent quantitative polymerase chain reaction (qPCR) analysis. According to the Oxford Community Stroke Project (OCSP) criteria mentioned above, the 6 AIS patients in the microarray group were classified as total anterior circulation infarction (TACI) in 1, partial anterior circulation infarction (PACI) in 4, and posterior circulation infarction (POCI) in 1. And the 39 AIS patients in the qPCR group were classified as 7 TACI, 27 PACI, and 5 POCI. The lncRNA expression of the AIS patients in different OCSP classification and CTRLs was compared.

### 3.2. Microarray Analysis Results

**Figure 1:**
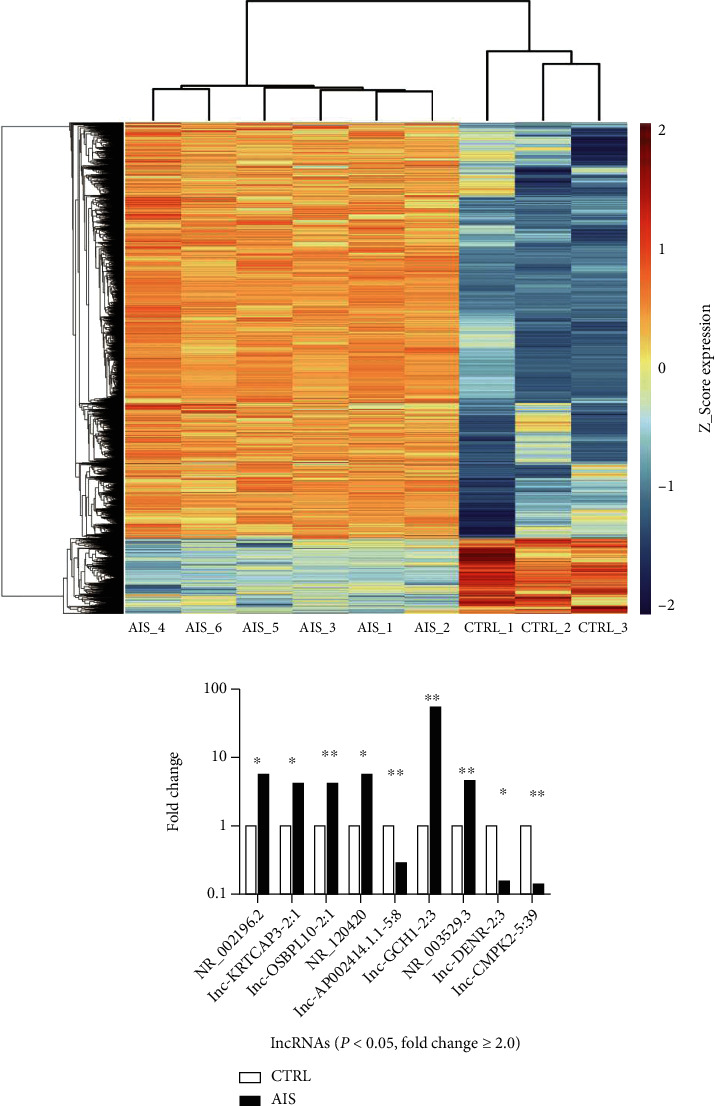
Heat map and hierarchical clustering for lncRNA profile comparison between the AIS patients and CTRLs and the microarray different expression profiles of nine lncRNAs which were selected for qPCR validation. (a) Each row represented one lncRNA, and each column represented one sample. The relative lncRNA expression was depicted according to the color scale. Red indicated upregulation; green indicated downregulation. 2, 1, 0, -1, and -2 were fold changes in the corresponding spectrum, whereas AIS represented acute ischemic stroke and CTRL represented controls. The differentially expressed lncRNAs were clearly classified into AIS and CTRL clusters. (b) The raw data of microarray was normalized with the quantile algorithm. Fold change as well as *P* value was calculated with normalized data. The heights of the columns in the figure represented the fold change. ^∗^*P* < 0.05 and ^∗∗^*P* < 0.01.

The expression levels of lncRNAs and mRNAs in blood were detected by microarray technology. As a result, 5686 lncRNAs and 1990 mRNAs with a fold change ≥ 2.0 in the expression profile were identified as the differentially expressed genes. A clustering heat map (Figure 1(a)) exhibited distinguishable expressed lncRNAs between AIS patients and CTRLs.

### 3.3. Validation of Differential lncRNA Expression by qPCR

**Figure 2:**
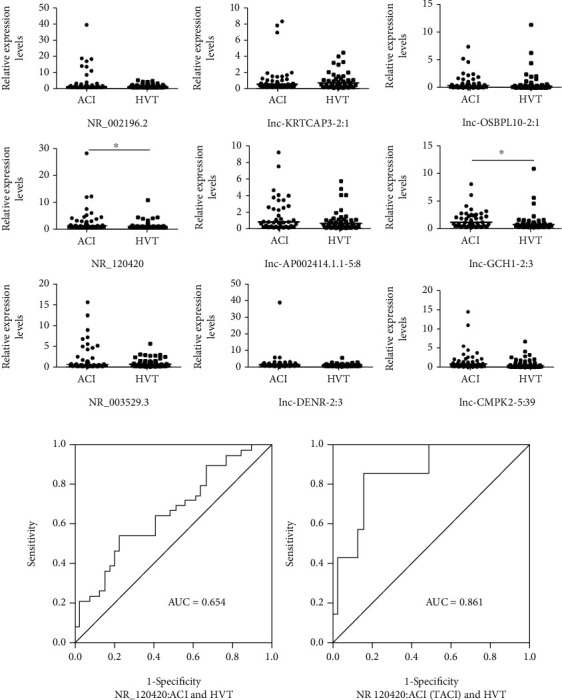
Relative expression levels of lncRNAs comparing the AIS and CTRL groups, measured by qPCR and ROC curve analysis for the lncRNA biomarkers. (a) The relative expression levels of nine lncRNAs in the AIS patients and CTRLs were verified by qPCR. The straight line in each figure represented the median. The original Ct value was treated with the 2^-*ΔΔ*CT^ method. ^∗^*P* < 0.05. (b) ROC curves compared the lncRNA biomarker expression levels of NR_120420 in blood between different AIS group and CTRL group. The diagonal in the figure was for reference.

Nine of the 5686 differently expressed lncRNAs identified by the microarray were selected to conduct qPCR validation. Six lncRNAs were upregulated (NR_002196.2, lnc-KRTCAP3-2:1, lnc-OSBPL10-2:1, NR_120420, lnc-GCH1-2:3, and NR_003529.3) and three lncRNAs were downregulated (lnc-AP002414.1.1-5:8, lnc-DENR-2:3, and lnc-CMPK2-5:39) in the microarray expression profile (Figure 1(b)). In the qPCR validation, the median of these nine lncRNAs in the AIS group was all higher than that in the CTRL group. Among the nine lncRNAs, NR_120420 and lnc-GCH1-2:3 showed a significant difference between the AIS and CTRL groups, while the differences were not statistically significant for the other lncRNAs (Figure 2(a)).

However, in the multivariate logistic regression analysis including patient clinical features and expression levels of NR_120420 and lnc-GCH1-2:3, only NR_120420 (OR 1.29, 95% CI 1.02-1.65, *P* = 0.037, *x*1), age (*x*2), and hyperlipidemia (*x*3) were found independently associated with the diagnosis of AIS. The regression model was established as follows: *Y* = 5.019 + 0.257*x*1 − 0.068*x*2 − 0.986*x*3 (Table 2).

### 3.4. NR_120420 Had High Specificity and Sensitivity in TACI Patients

A receiver operating characteristic (ROC) curve was drawn to detect the diagnostic accuracy of lncRNAs as biomarkers for AIS (Figure 2(b), left). The area under the curve (AUC) of NR_120420 was 0.65 (95% CI: 0.53-0.78, *P* = 0.019). At the optimal cutoff point (0.63), the sensitivity and specificity were 53.8% and 76.9%, respectively.

**Figure 3:**
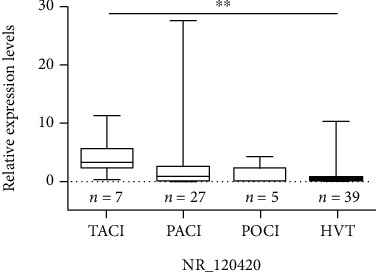
Comparison of NR_120420 expression in AIS patients with different OCSP classification to the CTRL group. The relative expression levels of NR_120420 in different classification were shown with minimums, the 25^th^ percentiles, medians, the 75^th^ percentiles, and maximums. The significance level (*α*) was adjusted to 0.016 (0.05/3) for multiple pairwise comparisons according to the Mann–Whitney test after overall comparisons of multiple groups according to the Kruskal-Wallis test, ^∗^*P* < 0.05 and ^∗∗^*P* < 0.01.

We further examined the expression levels of NR_120420 in different AIS subtypes (Figure 3). Compared to CTRLs, its expression was increased in TACI and PACI subtypes and decreased in the POCI subtype. The differences were significant only in the TACI subtype compared to CTRLs. A ROC curve (Figure 2(b), right) concerning NR_120420 in TACI patients showed the AUC reached 0.86 (95% CI: 0.73-0.99, *P* = 0.003). At the optimal cutoff point of 1.93, the sensitivity and specificity were 85.7% and 84.6%, respectively.

## 4. Discussion

In this study, we identified one specific circulating lncRNA (NR_120420) that exhibited significantly higher in the AIS patients, and the validation via qPCR showed NR_120420 has a high sensitivity and specificity in the diagnosis of total anterior circulation infarction.

By using microarray and qPCR, we successfully identified two circulating lncRNAs (NR_120420 and lnc-GCH1-2:3) significantly increased in AIS patients in the beginning. However, lnc-GCH1-2:3 was not found independently associated with the occurrence of AIS after balancing with patient baseline characteristics, which indicated lnc-GCH1-2:3 may be more likely associated with such baseline characteristics.

We found that NR_120420 showed a potential value in predicting the “TACI” AIS subtype; the sensitivity and the specificity reached 85.7% and 84.6%, respectively. Cerebral infarction volume of the patients with TACI is larger than that of the patients with PACI and POCI. More lncRNAs were released from injured brain cells and entered the blood flow via the disrupted blood-brain barrier in these patients. As a result, lncRNA in the peripheral whole blood of them would be significantly different from the CTRL group. The research of our group showed that NR_120420 knockdown inhibited apoptosis after cerebral infarction by downregulating the phosphorylation of a subunit of NF-*κ*B (P65) [18].

Recently, Wang et al. have also studied the role of another circulating lncRNA H19 in predicting AIS; they examined an AIS patient whose onset-to-admission time was less than 3 hours and reported that lncRNA H19 in plasma, lymphocytes, and neutrophils had a sensitivity and specificity of 0.806 and 0.920, 0.556 and 0.920, and 0.75 and 0.72 in predicting AIS, respectively [19]. Our result is comparable to theirs; however, we used the peripheral whole blood samples rather than different blood component, which may be faster in clinic. In addition, the included patients were different; our study included patients whose onset time was less than 6 hours while the aforementioned study included patients whose onset time was less than 3 hours. A recent study showed that the expression profile of whole blood lncRNAs after ischemic stroke changed significantly with time [16].

A limitation of our study was the small sample size for microarray analysis. In addition, this lncRNA microarray technology lacks the ability to unravel novel features of splice isoforms or fusion transcripts compared to RNA-seq technology. Besides, we will carry out investigation about the peripheral whole blood expression of this lncRNA in hemorrhagic patients in the future in order to clarify the exclusive role of this lncRNA for cerebral infarction and make our conclusion be more reliable and specific.

## 5. Conclusion

Our study indicated that NR_120420 could predict the total anterior circulation infarction with high sensitivity and specificity and hence could be potentially used as a biomarker for total anterior circulation infarction in AIS patients.

## Figures and Tables

**Table 1:** Clinical features of the AIS group and the CTRL group.

Features	AIS (*n* = 39)	CTRL (*n* = 39)	*P*
Age (years)	74 (15)	76.62 ± 10.34	0.134
Male/female (*n*/*n*)	15/24	21/18	0.173
Current smoking, *n* (%)	6 (15.4)	11 (28.2)	0.17
DM, *n* (%)	7 (17.9)	10 (25.6)	0.411
Hypertension, *n* (%)	23 (59.0)	17 (43.6)	0.174
Hyperlipidemia, *n* (%)	12 (30.8)	16 (41)	0.345
CHD, *n* (%)	7 (17.9)	4 (10.3)	0.329
Alcohol intake, *n* (%)	5 (12.8)	7 (17.9)	0.530
SBP (mmHg)	140.21 ± 19.86	130 (15)	0.480
DBP (mmHg)	82.82 ± 13.37	80 (13)	0.296
Heart rate (min^−1^)	80 (16)	76 (8)	0.149
Blood glucose (mmol/l)	7.1 (3.6)	6.9 (4)	0.889
TC (mmol/l)	4.32 ± 0.88	4.55 ± 0.96	0.273
TG (mmol/l)	1.45 (1.25)	1.27 (0.88)	0.232
LDL (mmol/l)	2.57 ± 0.70	2.57 ± 0.73	0.974
HDL (mmol/l)	1.15 (0.41)	1.2 (0.47)	0.678

DM: diabetes mellitus; CHD: coronary heart disease; SBP: systolic blood pressure; DBP: diastolic blood pressure; TC: total cholesterol; TG: triglyceride; LDL: low-density lipoprotein; HDL: high-density lipoprotein.

**Table 2:** Multivariate logistic regression.

Item	*B*	S.E.	Wald	df	*P*	OR	95% CI of OR	
							Lower limit	Upper limit
NR_120420	0.25	0.12	4.36	1	0.037	1.29	1.02	1.65
Age	-0.068	0.028	5.77	1	0.016	0.93	0.88	0.99
Hyperlipidemia	-0.99	0.55	3.18	1	0.075	0.37	0.13	1.10
Constant	5.02	2.17	5.35	1	0.021	151.3		

## Data Availability

The data of the microarray analysis are available from the Gene Expression Omnibus database (accession number: GSE102541) (https://www.ncbi.nlm.nih.gov/geo/query/acc.cgi?acc=GSE102541).

## Abbreviations

AIS: Acute ischemic stroke
qPCR: Quantitative polymerase chain reaction
OCSP: Oxford Community Stroke Project
CTRL: Control
ROC: Receiver operating characteristic
TACI: Total anterior circulation infarction
lncRNAs: Long noncoding RNAs
ncRNAs: Noncoding RNAs
PAC: Partial anterior circulation infarction
POCI: Posterior circulation infarction
AUC: Area under the curve
Ct: Cycle threshold
ANOVA: Analysis of variance
SD: Standard deviation
DM: Diabetes mellitus
CHD: Coronary heart disease
SBP: Systolic blood pressure
DBP: Diastolic blood pressure
TC: Total cholesterol
TG: Triglyceride
LDL: Low-density lipoprotein
HDL: High-density lipoprotein.
